# Editorial: Novel Psychoactive Drugs—The Saga Continues…

**DOI:** 10.3389/fnins.2021.650518

**Published:** 2021-02-25

**Authors:** Aviv M. Weinstein, Liana Fattore

**Affiliations:** ^1^Department of Behavioral Science, Ariel University, Ariel, Israel; ^2^Institute of Neuroscience-Cagliari, National Research Council of Italy, Cagliari, Italy

**Keywords:** novel psychoactive substances (NPS), drug intoxications, synthetic cannabinoids, synthetic cathinones, synthetic opioids, ayahuasca, piperazines, phenylethylamine & phenylalkylamines

This Research Topic has been planned, organized, and edited as a follow-up of the first Frontiers Research Topic on Novel Psychoactive Substances (NPS, https://www.frontiersin.org/research-topics/5249/novel-psychoactive-drugs) that by the end of 2020 has collected almost 134,000 visits. This 2nd Research Topic collects 15 articles, namely 11 original articles (four animal and seven human studies) and four reviews, and covers the main classes of NPS, including “old” drugs with renewed interest, such as ayahuasca.

Among the wide world of NPS, synthetic cannabinoids and cathinones continue to be among the most widely used NPS worldwide. In a preclinical study, Bilel et al. provide a pharmacological and behavioral characterization of the effects of a new *synthetic cannabinoid* belonging to the 3rd generation, AKB48 [APINACA, N-(1-adamantyl)-1-pentyl-1H-indazole-3-carboxamide], in rats. Besides the classic “tetrad,” the battery of behavioral tests included motor, sensorimotor, neurochemical and cardiorespiratory responses, place preference conditioning, and pre-pulse inhibition tests. All behavioral and neurochemical effects were fully prevented by the selective cannabinoid CB1 receptor antagonist/inverse agonist AM251 and blood concentrations of AKB48 were monitored and correlated with behavioral measurements. In two different clinical studies, Cohen, Mama et al. have first assessed the performance on executive function and emotional processing tasks in chronic *synthetic cannabinoids* users by using computerized neurocognitive function tests (i.e., the N-back task, Go/No-Go task, Wisconsin Sorting Card-like Task, and emotional face recognition task) and questionnaires of depression, anxiety and schizotypal traits and symptoms. The authors have reported significant impairments in mental flexibility and executive functions in synthetic cannabinoids users, along with elevated depressive and anxiety symptoms and schizotypal traits. Secondly, Cohen, Rosenzweig et al. have explored schizotypy measures and the personality characteristics of chronic synthetic cannabinoids users. They were reported to differ from natural cannabis users and non-users on dimensions of specific personality traits and schizotypy measures that may indicate psychotic proneness. Specifically, synthetic cannabinoids users have displayed higher scores of neuroticism and lower scores of agreeableness and extraversion compared with natural cannabis users and non-users, and lower levels of conscientiousness relative to non-users.

In a very elegant study, Zwartsen et al. combined integrated measurements (microelectrode arrays recordings of neuronal activity) with single target assays (monoamine reuptake transporter inhibition) to investigate the acute effects of several *synthetic cathinones* [4-methylethcathinone (4-MEC), 3-methylmethcathinone (3-MMC), 4-MMC, methylone, pentedrone, a-pyrrolidinovalerophenone (α-PVP), and 3,4-methylenedioxypyrovalerone (MDPV)]. The authors showed that inhibition of monoamine reuptake transporters is the most sensitive target for this set of synthetic cathinones, although the inhibition of neuronal activity at concentrations relevant for recreational exposure suggests additional targets for some cathinones. As many other NPS, synthetic cathinones are often used concomitantly with other drugs, which can lead to acute toxicities and fatalities. Kapitány-Fövény et al. compared opioid dependent patients under opioid substitution treatment with and without a history of synthetic cathinones use during therapy and found that use of synthetic cathinones during opioid substitution treatment (i) was associated with poorer treatment outcomes (including less adaptive strategies to cope with negative life events) and enhanced risk for reduced treatment retention, and (ii) might be characterized by more severe psychiatric symptoms and amotivation to change substance use among opioid dependent patients. In a first article, Papaseit, Pérez-Mañá et al. focused on the interactions between the synthetic cathinone mephedrone (4-methylmethcathinone, 4MMC) and alcohol, i.e., the most common two-drug combination reported among NPS recreational users in nightclubs, music festivals, and rave parties. The authors have found that alcohol increases the cardiovascular effects and abuse liability of mephedrone while mephedrone reduced the drunkenness and sedation produced by alcohol. Importantly, their combination induced a more intense feeling of euphoria and well-being in comparison to the two drugs alone.

In a second contribution, Papaseit, Olesti et al. conducted an observational study in a real-life setting of recreational use to assess pharmacokinetics and acute effects of 2,5-dimethoxy-4-ethylphenethylamine (2C-E), a psychedelic *phenylethylamine* with a chemical structure similar to mescaline. The authors have described severe alterations in perceptions, hallucinations, and euphoric-mood, even at low-moderate doses, which display high inter-individual variability and marked similarities with the psychedelic-like effects of other serotonin-acting drugs. Another synthetic phenethylamine with psychedelic and entactogenic effects, i.e., the compound 4-iodo-2,5-dimethoxy-N-(2-methoxybenzyl)phenethylamine (25I-NBOMe) better known as “N-Bomb,” was the object of a preclinical study by Miliano et al., which has described how this NPS alters the brain dopaminergic transmission, induces sensorimotor modifications, impairs the startle amplitude, and inhibits the prepulse inhibition in rats.

The role of dopamine in the molecular and neurobiological responses to stimulant NPS has been elegantly investigated by Loi et al. that used a combined *in vitro, in vivo*, and *in silico* approach to investigate the central mechanisms of action of desoxypipradrol, also known as 2-diphenylmethyl*piperidine* (2-DPMP). The study has demonstrated that 2-DPMP is a potent stimulant that directly interacts with the brain's rewards system mainly through molecular rearrangements toward an outward-facing conformation of the presynaptic dopamine transporter (DAT), which suggested a cocaine-type effect. Using the open-web crawling/navigating software NPS.Finder®, Arillotta et al. have identified a large number (426) of unknown *opioids* with a possible recreational/misuse potential, including 176 very potent fentanyl analogs not listed in either International or European NPS databases. The study not only highlights a strong interest by psychonauts toward opioid drugs, but also confirms the utility of psychonaut fora/platforms to better understand the online situation regarding opioids use and misuse. As last original article contribution, the double-blind, randomized and placebo-controlled clinical trial by Zeifman et al. have examined for the first time the impact of ayahuasca (a brew containing N,N-dimethyltryptamine and beta-carboline alkaloids) on suicidality in individuals with treatment-resistant depression, to test their hypothesis that ayahuasca would lead to decreases in suicidality, and reported promising, although limited and mixed, results.

In the first of the four reviews, Zawilska et al. have provided a comprehensive and updated overview of the history of NBOMe derivatives, a specific set of psychedelic *phenylalkylamines*, their central and peripheral effects, pattern of use and metabolism. Commonly observed adverse effects were reported and cases of non-fatal and lethal intoxications involving these compounds were discussed. Importantly, being the analysis of NBOMes in biological materials particularly challenging, the analytical methods most commonly used for detection and identification of NBOMes and their metabolites were presented. Then, Donnadieu-Rigole et al. described complications related to drugs used to improve sexual performance and/or to promote disinhibition (a phenomenon better known as “*chemsex*”), showing how use of these drugs can be dramatically associated with high-risk sexual behaviors. Specifically, the authors have reviewed complications related to the use of cathinones, methamphetamines, gamma-butyrolactone/gamma-hydroxybutyrate (GBL/GHB), ketamine, cocaine and speed in parties, sex meetings and homosexual/heterosexual practices, soliciting specific prevention, and intervention strategies. In their systematic review, Segawa et al. have provided an overview of Virtual Reality (VR), i.e., head mounted devices, in the assessment of cue reactivity (craving, psychophysiological response, and attention to cue) and treatment of addictive disorders, i.e., intervention in nicotine, cocaine, alcohol and cannabis addiction, and gambling. Current evidence suggests the VR provides benefits in the assessment and treatment of substance use disorders and gambling. Yet, contrary to craving provocation in VR that is effective across addiction disorders, treatments based exclusively on virtual exposure to drug related cues showed heterogeneous results. Finally, the review by Orsolini et al. examined the main clinical and psychopathological features of the psychoses induced by synthetic cannabinoids and cathinones and their therapeutic strategies, and underlie the main differences with the “classical” psychoses. Importantly, the authors have provided further insight on therapeutic strategies and practical guidelines for managing patients affected with synthetic/chemical NPS-induced psychoses.

Overall, we feel that the present Research Topic provides an interesting and valuable picture of the current state-of-the-art in the field that contributes to our understanding of the pharmacological and toxicological effects of NPS and their evolution on the market, and will likely help clinicians and emergency staff in managing intoxications symptoms.

## Author Contributions

AW and LF contributed equally to this Editorial of the 2nd Research Topic on NPS that they edited in 2020. Both authors contributed to the article and approved the submitted version.

## Conflict of Interest

The authors declare that the research was conducted in the absence of any commercial or financial relationships that could be construed as a potential conflict of interest.

